# Simultaneous Removal of Organic Pollutants and Pathogens from Stormwater by an Enhanced Ecological Gabion

**DOI:** 10.3390/toxics14030247

**Published:** 2026-03-12

**Authors:** Shuhui Gao, Pingping Li, Zizheng Zhao, Luobin Zhang, Kui Huang, Xiaojun Chai

**Affiliations:** 1 School of Environment and Municipal Engineering, Lanzhou Jiaotong University, Lanzhou 730070, China; 2 Gansu Province Key Laboratory of Yellow River Water Environment, Lanzhou Jiaotong University, Lanzhou 730070, China; 3 Water Resource and Hydropower Investigation and Design & Research Institute of Gansu Province, Lanzhou 730000, China

**Keywords:** ecological gabion, organic pollutants, pathogens, riparian zone, runoff

## Abstract

Stormwater runoff represents a significant vector for the transport of organic pollutants and pathogens into aquatic ecosystems, posing serious environmental and public health risks. Although extensively employed for bank stabilization, traditional gabion structures demonstrate constrained efficacy in pollutant removal. In this study, an enhanced ecological gabion (EG) system was developed by integrating a stratified configuration of functional fillers (ceramsite, maifanite, and biochar) with vegetation (*Iris germanica*). This design leverages synergistic effects to enhance the concurrent removal of dissolved organic matter (DOM), particulate organic matter (POM), and fecal indicator bacteria (FIB) from simulated stormwater. The system was evaluated in continuous flow experiments through comparison with a traditional gravel gabion (TG). Results showed that, compared with the TG, the EG exhibited markedly enhanced removal performance, with chemical oxygen demand (COD), NH_4_^+^–N, and TN removal efficiencies being approximately 2.48, 3.68, and 3.56 times those of the TG, respectively. In addition, the EG exhibited significantly higher removal efficiencies for both particulate organic carbon (POC) and dissolved organic carbon (DOC) than the TG, with increases of 329% and 137%, respectively. Fluorescence spectroscopy and particle size distribution analyses revealed that the EG effectively transformed and removed diverse DOM components and fine particulates. The stratified filler media synergistically enhanced pollutant retention, with biochar serving as the primary agent for nutrient and pathogen adsorption. These findings demonstrate the viability of the EG as an integrated, eco-friendly solution for enhanced stormwater purification in riparian zones, providing a compact and multifunctional alternative to conventional end-of-pipe systems.

## 1. Introduction

Stormwater runoff is an integral component of the regional hydrological cycle. However, it also represents a major pathway for the transport of pollutants into aquatic ecosystems. As runoff travels over urban and agricultural surfaces, it mobilizes and transports a complex mixture of pollutants, including nutrients, particulate matter, organic contaminants, and pathogenic microorganisms into rivers and lakes [1]. Climate change amplifies this challenge by intensifying hydrological extremes, which in turn elevates the frequency and severity of pollution events [2]. The first flush is characterized by a sharply elevated pollutant load, including concentrated organic matter and pathogens, from the initial phase of runoff. Documented concentrations in initial stormwater runoff show that chemical oxygen demand (COD) and fecal indicator bacteria (FIB; e.g., *Escherichia coli*) can exceed 10^6^ mg/L and 10^4^ CFU/100 mL, respectively [3,4]. This type of pollution significantly degrades water quality, posing immediate and severe threats to both human health and aquatic ecosystems. Consequently, it is essential to implement effective interception and mitigation strategies to prevent contaminants from entering receiving water bodies through surface runoff.

Mitigation strategies for stormwater pollution encompass a systematic approach, spanning source control, process mitigation, and end-of-pipe treatment. The diffuse spatial footprint and scattered nature of pollution sources limit the effectiveness of conventional engineered solutions that rely on centralized treatment. Moreover, the efficiency of process mitigation facilities is highly dependent on rainfall characteristics. The combined effect of sharply increased hydraulic loading and reduced retention time during storm events compromises treatment performance and poses a risk of secondary pollution through the remobilization of retained contaminants [5]. Thus, end-of-pipe measures serve as the final critical barrier to prevent contaminants from reaching sensitive waters. Constructed wetlands can provide centralized treatment prior to discharge; however, their large spatial requirement and the scarcity of suitable land often limit widespread application. In contrast, bioretention systems, as prevalent small-scale end-of-pipe technologies, have demonstrated high removal efficiencies for conventional pollutants such as total phosphorus (TP) and total nitrogen (TN) [6]. However, the performance of these end-of-pipe measures in removing dissolved organic matter (DOM) and pathogens is often inconsistent and limited. Reported removal efficiencies typically range from approximately 20–40% for DOM components and less than a 1-log reduction for pathogens [7,8]. Therefore, it is imperative to develop a compact and efficient treatment approach that can be implemented within riparian zones to achieve substantial reductions in pollutants such as DOM and pathogenic bacteria present in runoff.

Riparian zones, functioning as vital interfaces between terrestrial and aquatic ecosystems, serve as natural filters that enhance riverine water quality by mitigating pollutants originating from surface runoff [9]. However, in engineered environments where natural buffers are absent or degraded, structures such as gabion revetments are commonly employed for bank stabilization and erosion control. Traditional gabion revetments, typically filled with conventional rock materials, demonstrate limited capacity for pollutant attenuation due to the low adsorption potential of the stone fill [10]. In response, recent engineering approaches have aimed to improve these structures by optimizing stone packing, incorporating vegetation, and promoting microbial colonization, thereby transforming them into multifunctional “ecological gabions” [11,12]. A key factor in this evolution is the selection of filler media, which is increasingly recognized as paramount for effective non-point source pollution interception [3]. For example, in a comparative analysis of filler materials, Wang et al. [13] reported that pebbles achieved the highest NH_4_^+^-N removal efficiency (65.26%), demonstrating superior performance over granite and construction waste blocks. As a complementary approach, prefabricated improved eco-concrete revetments, designed for rapid seepage and high permeability, achieved mean removal efficiencies of 64% (NH_4_^+^–N), 51% (TP), and 42% (COD), respectively [14]. These results indicate that intentionally combining ceramsite (for physical filtration), maifanite (for ion exchange), and biochar (for high adsorption capacity) can produce a synergistic treatment system for removing a broad spectrum of runoff pollutants. However, despite the potential of such multi-media configurations, systematic studies providing a comprehensive assessment of enhanced EGs for the concurrent removal of a broad pollutant spectrum (including diverse organics and pathogens) from stormwater remain limited. This knowledge gap underscores the need for comprehensive, integrated studies on the design and operational performance of next-generation gabion systems.

To close this knowledge gap, this study designed an enhanced EG composed of stratified functional fillers (ceramsite, maifanite, and biochar) integrated with conventional gravel to evaluate pollutant removal performance. The specific objectives of this study were to (1) quantitatively evaluate the performance of the enhanced EG in removing DOM, particulate organic matter (POM), and FIB from synthetic stormwater relative to a traditional gabion (TG), and (2) elucidate the underlying removal mechanisms by examining pollutant distribution on filler surfaces, fluorescence characteristics of DOM, and particle-size distributions of suspended solids. DOM was characterized using three-dimensional excitation–emission matrix (3D-EEM) fluorescence spectroscopy, POM was analyzed with a laser particle-size analyzer, FIB was quantified by quantitative real-time polymerase chain reaction (qPCR) and pollutant distribution on filler surfaces was examined. Collectively, this work aims to provide a scientific basis for developing simple, cost-effective, green infiltration technologies to enhance stormwater quality control at the riparian interface.

## 2. Materials and Methods

The simulated runoff was prepared from a soil matrix obtained from floodplain deposits adjacent to the Zhongshan Bridge in Chengguan District, Lanzhou City, China. The physicochemical properties, such as pH, electrical conductivity, ammonia nitrogen, total nitrogen, total phosphorus, moisture content, and organic matter content, are summarized in Table 1. A soil-deionized water mixture at a 1:100 ratio was stirred at 300 rpm for 30 min using a magnetic stirrer, followed by a 30-min settling period to simulate the characteristics of first-flush runoff. The ceramsite, maifanite, biochar, and plant (*Iris germanica*) filters used in this study were obtained from the Heping Industrial Source Store in Yuzhong County, Lanzhou City, China. The EG devices were fabricated by the Yusheng Industrial Co., Ltd., located in the Chengguan District of Lanzhou City, China.

### 2.1. Experimental Setup

**Figure 1:**
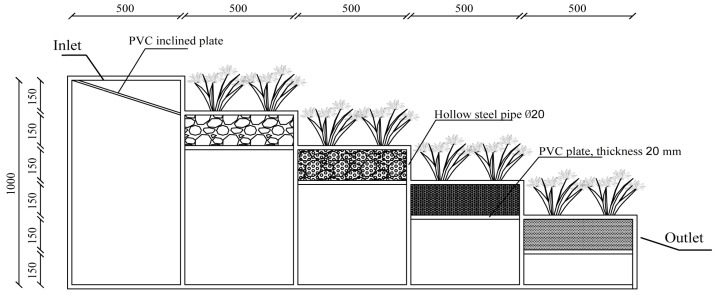
Schematic diagram of the ecological gabion.

As shown in Figure 1, the EG apparatus is constructed from stainless steel and comprises four layers of filtration media. The first layer of cobblestones provided structural support and distributed flow. The second layer, composed of cobblestones and ceramsite, enhanced physical filtration. The third layer, made of cobblestones and maifanite, adsorbed dissolved organic matter (DOM). The fourth layer combined biochar with cobbles and maifanites, with the high specific surface area of biochar facilitating advanced removal of organic pollutants. Plants were grown on each terrace at a density of 45 plants·m^−2^ to enhance pollutant uptake by their root systems and to stimulate microbial growth for enhanced degradation. A continuous-flow regime was used; for each experimental cycle, 10 L of synthetic stormwater was applied to the system. The influent was introduced naturally onto the inlet distribution plate (set at a 5% slope) at a controlled flow rate of 400 mL/min. Effluent from both the TG and EG systems was collected in clean plastic basins. Subsequently, a 400 mL aliquot of each sample was transferred into polyethylene bottles and immediately stored at 4 °C prior to laboratory analysis. For the experiment, independently prepared water samples were used, with water inflow conducted at two-day intervals for a total of ten cycles. The experiment was conducted at Lanzhou Jiaotong University from October to November 2024, where the ambient temperature ranged from 23 to 26 °C.

### 2.2. Physicochemical Analysis

The determination of physicochemical properties followed the methods of [15]. Electrical conductivity (EC) of the supernatant was measured with an electrical conductivity meter (DDS-307; Leici, Shanghai, China). The mixture was then filtered through a 0.45 μm membrane, diluted tenfold, and the filtrate was used to determine dissolved organic carbon (DOC) using a TOC analyzer (Multi N/C 2100; Analytik Jena, Jena, Germany). The remaining filtrate was analyzed for NH_4_^+^–N, NO_3_^−^–N, total nitrogen (TN), and total phosphorus (TP) using a UV spectrophotometer (UV-3100; Mapada, Shanghai, China), with absorbance measured at 410 nm (NH_4_^+^–N), 220 nm (NO_3_^−^–N), and 275 nm (TN and TP) [3].

### 2.3. Propidium Monoazide (PMA) Treatment, Deoxyribonucleic Acid (DNA) Extraction, and qPCR Procedures

For solid samples, 1 g of fresh material was added to 10 mL of phosphate buffer (0.01 mol·L^−1^, pH 7.4) and stirred magnetically at 300 rpm for 30 min. Subsequently, 2 mL of the mixture was transferred into a 2-mL centrifuge tube. For liquid samples, an appropriate volume was centrifuged at 8000× *g* (gravitational acceleration) for 5 min to pellet the precipitate, which was then transferred to a 2-mL centrifuge tube. PMA was used to selectively identify and suppress amplification from dead cells and extracellular DNA. Upon photoactivation, PMA cross-links to DNA in membrane-compromised cells and free DNA, rendering them non-amplifiable in subsequent PCR. This step ensured that the resulting PCR and sequencing data predominantly reflected DNA derived from viable cells [16]. A 5-µL aliquot of PMA (20 mmol·L^−1^) was added to the 2-mL mixture to achieve a final concentration exceeding 40 µmol·L^−1^. After thorough mixing, the solution was incubated at 4 °C for 10 min, followed by 20 min of photolysis using a light-emitting diode (LED) photolysis device (EM200, Takara, Kusatsu, Shiga, Japan). The tubes were inverted every 5 min during incubation to promote uniform contact between PMA and DNA from non-viable cells. Following PMA treatment, total DNA was extracted using the TIANamp Soil DNA Kit (Tiangen Biotech, Beijing, China). DNA concentration was quantified using a Qubit 4 Fluorometer (Thermo Fisher Scientific, Waltham, MA, USA) to verify adequate yield for downstream qPCR analysis. Quantitative PCR (qPCR) was performed on a Thermal Cycler Dice Real Time System Lite (Takara, TP700, Shiga, Japan) following the protocol described by Duan et al. [17]. Reaction setup, cycling conditions, and quantification methods were also adopted from this reference. The quantification of bacterial 16S rRNA, total coliform 16S rRNA, fecal coliform 16S rRNA, and *Enterococcus* 23S rRNA was performed using the SYBR Green dye-based method, whereas the *Escherichia coli* tnaA gene was quantified with the TaqMan probe-based approach. All primer and probe sequences, such as total coliforms, fecal coliforms, *Escherichia coli* and *Enterococcus* spp., are listed in Table 2 and were synthesized by Sangon Biotech (Shanghai, China) Co., Ltd. (Shanghai, China) Standard curves were generated using ten-fold serial dilutions of plasmids carrying the target genes, which were cloned into the pMD20-T vector (Takara, Dalian, China), with detailed preparation procedures described by [18].

### 2.4. Analysis of Spectrometric Determination

A water sample (100 mL) was first passed through a 0.45 μm glass fiber filter to isolate the DOM fraction. To recover POM, the retained material on the 0.45 μm membrane was transferred into 10 mL of 0.1 M NaOH and extracted in the dark at 4 °C for 24 h [21]. Following extraction, the solution pH was carefully readjusted to that of the original sample. The resulting extract was filtered through a 0.22 μm membrane to obtain the POM fraction [22]. Both DOM and POM fractions were determined using a TOC/TN analyzer (Multi N/C 2100, Analytik Jena, Jena, Germany) to quantify DOC and POC concentrations, respectively.

Suspended particulates were collected from a separate 100 mL sample with centrifugation at 4000 rpm for 30 min. The recovered pellet was freeze-dried and subjected to density fractionation to characterize POM particle size distribution, using a laser diffraction analyzer (Mastersizer 3000, Malvern Panalytical, Malvern, UK) [23]. Three-dimensional excitation–emission matrix (EEM) fluorescence spectra of DOM were measured using a fluorescence spectrophotometer (F-7100, Shimadzu, Kyoto, Japan). The photomultiplier tube voltage was set to 700 V. Excitation wavelengths ranged from 220 to 450 nm, while emission wavelengths scanned from 220 to 550 nm. Both excitation and emission slit widths were maintained at 5 nm, with a scanning speed of 12,000 nm min^−1^. To minimize inner-filter effects, samples were diluted to 3 mg L^−1^ prior to measurement following previously reported procedures [24]. Background spectra of Milli-Q water (Merk Millipore, Burlington, MA, USA) were recorded before each analysis. All fluorescence data were corrected for baseline deviation, smoothed, and exported for further processing as described in [25].

### 2.5. Multivariate Statistical Analysis

Schematic diagrams were drawn using AutoCAD 2024. Contour maps were generated in Origin 2021, while bar charts and box plots were created using GraphPad Prism 9.5.0. Statistical differences between samples were evaluated using a *t*-test or one-way analysis of variance (ANOVA). Prior to analysis, data normality was assessed using the Shapiro–Wilk test, and homogeneity of variance was tested using Levene’s test. When data did not meet these assumptions, non-parametric tests (Mann–Whitney, Kruskal–Wallis test) were applied. The distribution characteristics of percentage data were also examined prior to analysis. Significance levels were set at * *p* < 0.05, ** *p* < 0.01, and *** *p* < 0.001. Spearman’s rank correlation analysis was performed using RStudio (version 4.3.1) and presented as quadrant correlation plots.

## 3. Results

### 3.1. Water Quality Comparison: Traditional vs. Ecological Gabions

**Figure 2:**
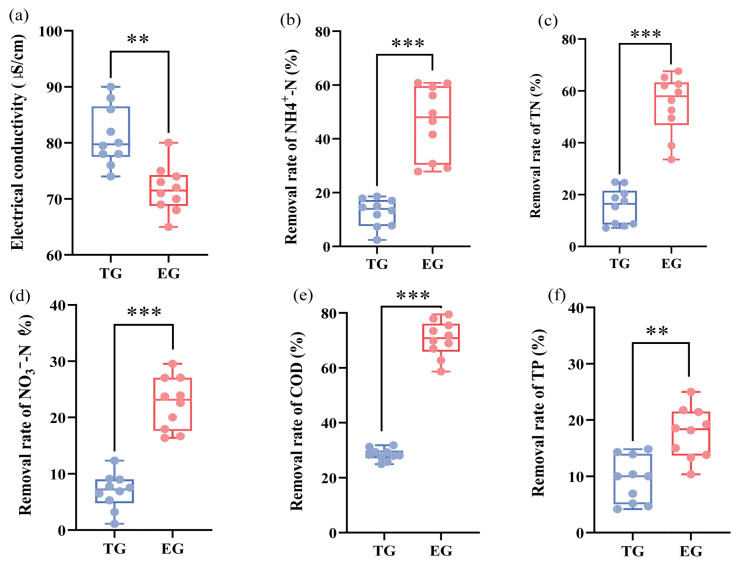
Removal rate of physicochemical pollutants in effluent of traditional gabion (TG) and ecological gabion (EG). (**a**) Electrical conductivity; (**b**) Removal rate of NH_4_^+^–N; (**c**) Removal rate of TN; (**d**) Removal rate of NO_3_^−^–N; (**e**) Removal rate of COD; (**f**) Removal rate of TP. ** and *** mean *p* value less than 0.01 and 0.001, based on the statistical significance using *t*-test.

As shown in Figure 2a, the average electrical conductivity was 71.70 μS·cm^−1^ for the EG effluent, compared with 81.15 μS·cm^−1^ for the TG effluent. These results indicate that EG exhibited a modestly enhanced capacity to reduce ionic concentrations, likely owing to improved adsorption and biogeochemical processes within the optimized filler matrix. As shown in Figure 2b, EG achieved an NH_4_^+^–N removal efficiency ~4.67 times that of TG, likely due to the optimized filler matrix that enhances microbial colonization and supports nitrification [26]. The effective removal of NH_4_^+^–N served as the primary driver for the notable reduction in TN within the EG system. The EG system exhibited an average removal efficiency 33.6% higher than that of the TG system (Figure 2c), highlighting the crucial role of bioavailable nitrogen conversion. In contrast, NO_3_^−^–N removal remained limited in both systems, although EG and TG showed relative mean advantages of 23.6% and 6.8%, respectively (Figure 2d). This limitation likely stems from a fundamental constraint of gabion structures: their predominantly aerobic conditions hinder the anoxic environments required for efficient denitrification [27]. The modest improvement observed in the ecological units is likely attributable to localized anoxic microsites within the finer filler material [28].

For organic matter removal, the EG system demonstrated high efficacy, with a COD removal rate of 70.5% that substantially exceeded the 42.1% achieved in the traditional system (Figure 2e). This result confirms the establishment of an active microbial community capable of degrading organic pollutants under the alternating micro-aerobic/anoxic conditions promoted by the complex filler geometry [29]. In contrast, phosphorus removal remained relatively low, with rates ranging from 10% to 25% in EG and 4% to 15% in TG (Figure 2f). This result suggests that removal was attributable primarily to physical sedimentation, as the fillers (e.g., maifanite, biochar) exhibited limited adsorption capacity for dissolved phosphate [30]. These findings indicate that effective long-term phosphorus removal necessitates the integration of high-capacity adsorbents [31]. As demonstrated, the EG system exhibited high efficacy in removing NH_4_^+^–N, TN, and COD, owing to its enhanced filtration units and selected plant species. However, removal efficiencies for NO_3_^−^–N and TP were relatively limited, indicating the need for targeted design improvements. Potential strategies include promoting anoxic/micro-anoxic niches to enhance denitrification (e.g., by adjusting hydraulic retention time or filler geometry) and incorporating high-capacity phosphorus adsorbents or reactive media to secure long-term TP retention.

### 3.2. Removal of Particulate and Dissolved Organic Carbon

#### 3.2.1. Removal of Total Contents of POC and DOC

**Figure 3:**
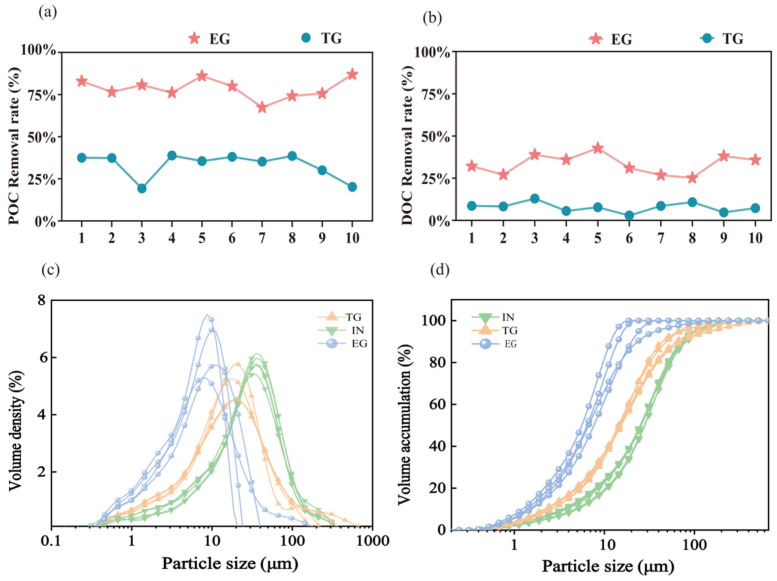
Removal rates of DOC (**a**) and POC (**b**) in gabion effluent and their particle size frequency distribution (**c**) and cumulative distribution (**d**). TG and EG represent traditional gabion and ecological gabion, respectively. IN means influent of the gabion.

As shown in Figure 3, the EG system exhibited superior organic carbon removal, with a marked divergence between POC and DOC responses. POC was retained or removed far more effectively than DOC, suggesting dominant physical/biological retention mechanisms for POC and limited DOC attenuation. The EG system achieved higher POC removal rates of 67.38–86.95%, significantly outperforming the TG system with removal rates of 19.30–38.85% (Figure 3a). Additionally, the EG removal rates of DOC (25.18–42.72%) were moderate but substantially higher than those of TG (2.96–12.97%) (Figure 3b). This contrast highlights the enhanced design of EG, which effectively addresses both particulate and dissolved forms of organic pollutants commonly present in stormwater runoff. These results underscore the EG’s high efficacy in controlling stormwater organic pollution, especially for particulate matter.

The efficient removal of POC in the EG system is driven primarily by physical filtration and interception within its multi-layer filler matrix (e.g., biochar, maifanite), which effectively captures large organic particles. In contrast, DOC removal entails more complex processes, including adsorption onto high-surface-area fillers followed by biodegradation via the established biofilm community [26,32]. The moderate DOC removal efficiency observed in the EG system indicates that, although adsorption can capture a portion of dissolved organics, complete mineralization depends on microbial activity, which is likely influenced by factors such as hydraulic retention time and the bioavailability of DOC compounds [33].

#### 3.2.2. Removal of DOC Components

**Figure 4:**
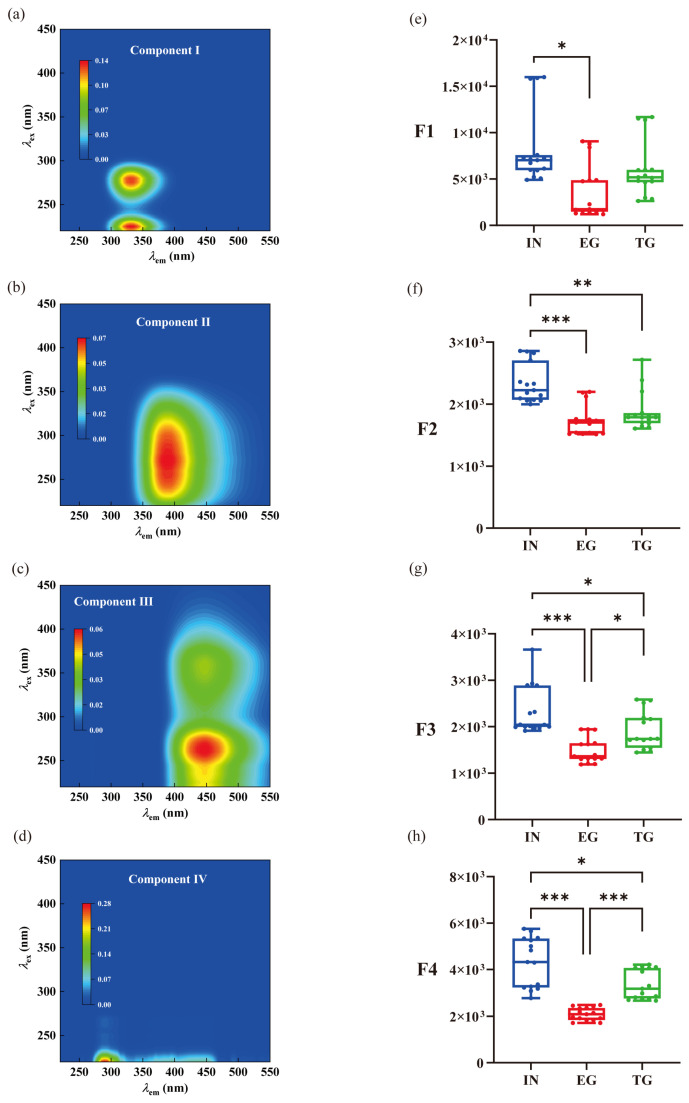
Contour plots of the 4 fluorescent components (**a**–**d**) identified by PARAFAC, and F-max plots (**e**–**h**), respectively. TG and EG represent traditional gabion and ecological gabion, respectively. IN means influent of the gabion. *, **, and *** mean *p* value less than 0.05, 0.01, and 0.001, based on the statistical significance using one-way ANOVA.

Parallel Factor (PARAFAC) analysis of fluorescence EEMs provided deep insight into the transformation and source identification of DOM. Four distinct components were identified in the influent (IN): C1 (protein-like, associated with tyrosine/tryptophan), C2 (fulvic-like), C3 (humic-like), and C4 (microbial by-product-like) (Figure 4a–d).

Initial runoff flushes surface-deposited sediments and organic debris into water bodies, leading to an increase in component C1 concentration. As a representative protein-like substance, component C1 serves as a key indicator for monitoring water quality in drinking water sources. The marked decrease in C1 observed in the EG effluent (Figure 4e) highlights the effective microbial degradation of labile, bioavailable organic nitrogen compounds within the EG [34]. This process is critical for mitigating the release of nitrogenous nutrients that could drive eutrophication. Thus, compared to TG, EG demonstrated further reductions in fulvic-like and humic-like components by 7.76% and 22.35%, respectively (Figure 4f,g). The observed reduction suggests that removal may involve mechanisms beyond biodegradation, e.g., adsorption onto biochar or co-precipitation [35]. Storm events can increase the leaching of humic and fulvic acids from SOM. Once released into the aquatic environment, these humic substances generally demonstrate a high degree of stability. Regarding C4, these substances are primarily generated through the microbial degradation of high-molecular-weight organic matter. Their concentration often reflects the level of microbial activity within the aquatic environment. Notably, the C4 in EG exhibited a significantly higher removal rate of 37.92% compared to TG (Figure 4h). Consequently, the EG effluent exhibited a marked reduction in fluorescence intensity across all components, while the TG—lacking specialized fillers and associated microbial communities—showed minimal change in its DOM fluorescence signature, merely confirming its limited treatment capacity beyond simple particulate settling.

#### 3.2.3. Removal of POC Components

Analysis of particle size distribution (PSD), detailed in Figure 3c,d, offers critical insight into the distinct removal mechanisms of the EG and TG systems. The influent displayed a relatively broad PSD with a consistent median (D50) between 24.1 and 31.1 μm, reflecting a predominance of medium-sized organic particles in stormwater. In contrast, the EG effluent exhibited a pronounced shift toward finer particles: the average size dropped sharply to 6.49~12.36 μm, D50 values fell to 5.92~6.72 μm, and D90 declined dramatically to 11.2 μm from approximately 76 μm in the influent. This pronounced shift highlights the system’s high efficiency in retaining larger particles and suggests that, beyond simple physical sieving, the EG’s filler matrix (e.g., biochar, maifanite) promotes particle aggregation, bioflocculation, and deposition within its complex pore network [36]. Consequently, the effluent contained a stable population of fine particles that are less susceptible to conventional physical filtration. Conversely, the TG effluent exhibited a PSD largely unchanged from the influent, maintaining an average size of 22.71–37.59 μm and a D50 near 16.4 μm. The only notable change was a slight D90 reduction to 66.9–76 μm, reflecting a system capable of removing only the very largest particles—a performance consistent with dependence on gravitational settling and simple filtration within a uniform gravel matrix [37]. This minimal alteration underscores the TG’s lack of advanced processes for POM retention and transformation. Thus, while the EG performs as an efficient dynamic filter and bioreactor, the TG is limited to functioning primarily as a coarse sediment trap. The effective capture of larger particles in the EG is therefore a key factor in its high POC removal efficiency and in mitigating the release of particle-associated pollutants.

### 3.3. Removal of Fecal Indicator Bacteria (FIB)

**Figure 5:**
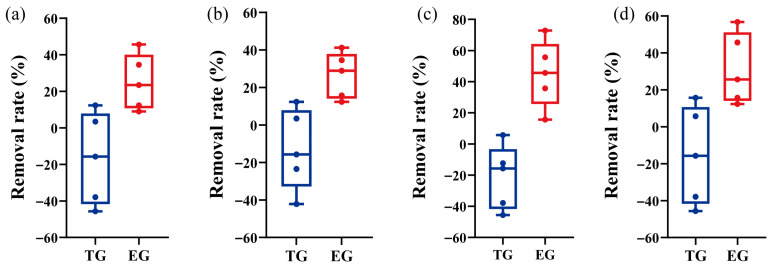
Removal rates of (**a**) Total coliforms, (**b**) Fecal coliforms, (**c**) *Escherichia coli*, and (**d**) *Enterococci* in the traditional gabion (TG) and ecological gabion (EG).

As shown in Figure 5, the comparative analysis revealed a significant disparity in the removal efficiency of active FIB between the TG and EG systems. The TG system displayed limited effectiveness, showing average net increases of 13% for total coliforms, 17% for fecal coliforms, 21% for *Escherichia coli*, and 15% for *Enterococcus* spp. The observed net increases imply the possible proliferation or release of these pathogens within the TG system. In contrast, the EG system exhibited markedly improved and consistent removal performance, achieving average efficiencies of 26%, 25%, 45%, and 31% for the respective bacterial indicators. These results collectively indicate that the EG system is effective in reducing FIB loads in stormwater, offering a promising mitigative strategy related environmental and public health risks.

In the EG system, the filter medium (e.g., biochar, gravel) enables the effective removal of pathogenic microorganisms, including bacteria and viruses. This is primarily attributed to physical filtration facilitated by the medium’s porous structure and optimized grain size distribution. Related research indicates that the concentration of pathogenic microorganisms correlates with the content of fine particles [38], suggesting that physical interception and enhanced particle sedimentation can substantially reduce pathogen levels in stormwater runoff. Soil particles smaller than 10 μm have been shown to effectively retain *Escherichia coli*. Moreover, the abundant oxygen-containing functional groups and surface charges on the filter media promote the effective adsorption of oppositely charged pathogenic microorganisms through electrostatic interactions, hydrophobic effects, and coordination bonds [39]. In addition, plants and indigenous microorganisms (e.g., actinomycetes and fungi) in bioretention systems secrete antibacterial compounds, including organic acids, phenolics, and lysozymes. These compounds inhibit the activity of pathogenic microorganisms, thereby collectively contributing to their reduction [40]. Research on *Escherichia coli* O157:H7 in constructed wetlands highlights key determinants of its survival. While Zhang et al. [41] identified ammonia nitrogen and available phosphorus as critical physicochemical factors, Sousa et al. [42] noted that indigenous microbial taxa, such as *Aeromonas*, also exert a considerable influence on *E. coli* persistence. Consequently, advancing EG design through optimized media selection and vegetation emerges as a key research direction to enhance its efficacy as a reliable, nature-based barrier, offering a robust strategy for mitigating waterborne pathogen dissemination.

### 3.4. Stratified Filtration Mechanisms and Synergistic Pollutant Removal in EG

The superior performance of the EG system can be attributed to the stratified, multifunctional design of its composite fillers—ceramsite, maifanite, and biochar—which operate synergistically to remove particulate and dissolved pollutants. This section clarifies the physiochemical mechanisms responsible for the removal of nutrients, organic matter, and microbial contaminants, based on analyses of surface characteristics, pollutant accumulation, and PSD.

#### 3.4.1. Surface Properties and Nutrient Retention

**Figure 6:**
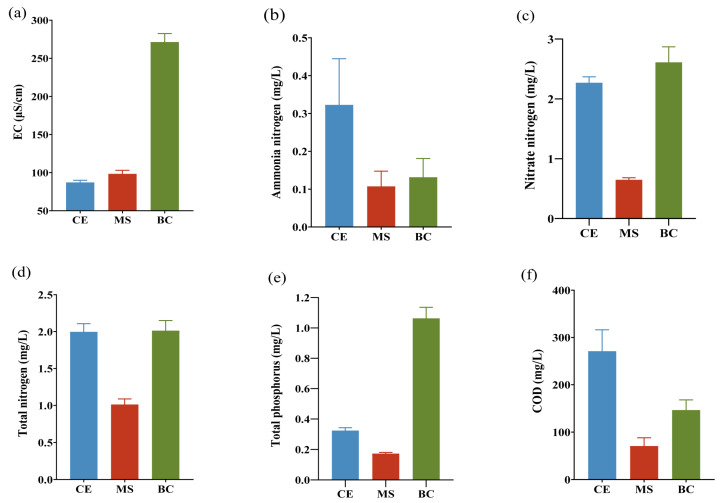
Changes in the physicochemical properties of filler surfaces in the ceramsite (CE), maifanite (MS), and biochar (BC) layers. (**a**) Electrical conductivity (EC); (**b**) Ammonia nitrogen concentration; (**c**) Nitrate nitrogen concentration; (**d**) Total nitrogen concentration; (**e**) Total phosphorus concentration; (**f**) Chemical oxygen demand (COD).

As shown in Figure 6a, biochar exhibited the highest surface conductivity (271 ± 3.5 μS·cm^−1^) among the three media, while maifanite and ceramsite showed significantly lower values. The high conductivity of biochar is correlated with its excellent capacity to adsorb soluble ions, owing to its abundant surface functional groups that electrostatically retain charged contaminants [43], thereby enhancing its overall pollutant-removal performance. Although maifanite also exhibited a considerable affinity for inorganic ions, its adsorption capacity remained lower than that of biochar. Furthermore, a marked divergence was observed in the nitrogen adsorption behaviors among the three media. Ceramsite exhibited the highest surface NH_4_^+^–N concentration (0.32 ± 0.02 mg·kg^−1^), followed by biochar (0.13 ± 0.01 mg·kg^−1^) and maifanite (0.11 ± 0.01 mg·kg^−1^) (Figure 6b). The pronounced NO_3_^−^–N enrichment on biochar is attributable to its porous texture and surface chemistry, which provide abundant sorption sites and can facilitate weak chemical interactions with nitrate ions [44]. Regarding TN, both biochar and ceramsite exhibited substantially higher surface concentrations—2.01 and 1.99 mg·kg^−1^, respectively—while maifanite showed the lowest concentration at 1.01 mg·kg^−1^ (Figure 6d). These results indicate that biochar and ceramsite exhibit stronger overall nitrogen retention than maifanite: biochar preferentially accumulates nitrate, while ceramsite favors ammonium retention. For TP, biochar also outperformed the other media, with a surface concentration of 1.06 mg·kg^−1^ (Figure 6e), approximately 3.3 and 6.2 times higher than those of ceramsite and maifanite, respectively. The high phosphorus uptake by biochar is attributable to its abundant surface functional groups, which enhance chemisorption of phosphate ions [45]. In contrast, maifanite exhibited limited phosphorus retention, likely relying primarily on physical adsorption with insufficient chemical complexation [46].

#### 3.4.2. Organic Matter Fractionation and Removal Pathways

**Figure 7:**
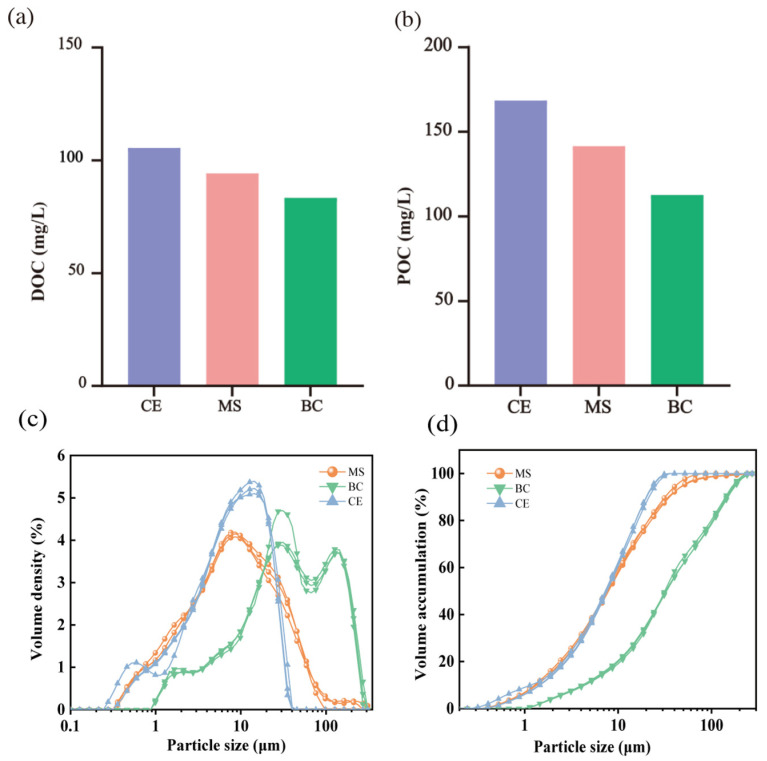
Concentrations of DOC (**a**) and POC (**b**) on the filler surface and their particle size distributions of frequency (**c**) and cumulative (**d**) in ceramsite (CE), maifanite (MS), and biochar (BC) layers.

Surface concentration measurements revealed a consistent descending order for both DOC and POC. Ceramsite demonstrated the highest accumulation, with DOC and POC concentrations of 105.51 and 168.42 mg·kg^−1^, respectively. This was followed by maifanite (94.27 and 141.41 mg·kg^−1^) and then biochar (83.40 and 112.59 mg·kg^−1^), as shown in Figure 7a,b. Primarily, biochar’s well-developed microporous structure promotes inward diffusion and adsorption of DOM into internal pores rather than accumulation on external surfaces, yielding lower surface-measured DOC concentrations [47]. Additionally, the layered configuration established a concentration gradient: the upper ceramsite layer encountered the highest pollutant load, while the lower biochar layer received pre-treated water with reduced DOM and POM availability, limiting its absolute surface accumulation despite its high intrinsic adsorption capacity. The functional differentiation is reflected in DOC:POC ratios of 0.74 for biochar, 0.67 for maifanite, and 0.63 for ceramsite. These values indicate that ceramsite has a greater propensity for particulate interception, whereas biochar shows a stronger relative affinity for dissolved fractions—consistent with the adsorption preferences imposed by their pore size distributions.

Particle size analysis of eluted surface particles corroborated the proposed removal mechanisms (Figure 7c,d). The retention profile of ceramsite was concentrated in the fine particle range, peaking at 14.5 µm with a maximum around 40 µm, which aligns with its role in intercepting colloidal and fine POM [48]. In contrast, maifanite captured a broader size spectrum, extending up to 310 µm. Biochar demonstrated the widest and a bimodal distribution, with peaks at approximately 31.1 µm and within the 127–144 µm range, confirming its superior, broad-spectrum particulate capture capacity facilitated by its complex porous network. This multi-modal retention underpins its key role in removing particle-bound pollutants and is characteristic of filter media with heterogeneous pore structures [49].

#### 3.4.3. Microbial Pathogen Sequestration at the Filler Interface

**Figure 8:**
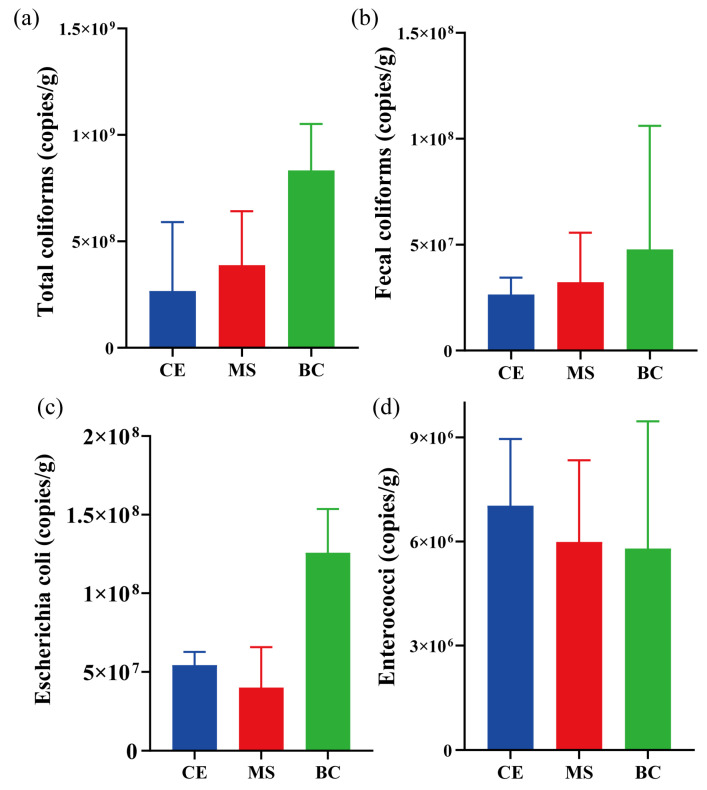
Concentration of fecal indicator bacteria on the surface of the filler of ceramsite (CE), maifanite (MS), and biochar (BC) layers. (**a**) Total coliforms; (**b**) Fecal coliforms; (**c**) *Escherichia coli*; (**d**) *Enterococcus* spp.

Results demonstrated that biochar had the strongest adsorption capacity for total coliforms, fecal coliforms, and *E. coli*, with surface abundances reaching 8.3 × 10^8^, 4.7 × 10^7^, and 1.2 × 10^8^ copies g^−1^, respectively (Figure 8)-significantly higher than those of ceramsite and maifanite. This superior performance is primarily due to biochar’s abundant oxygen-containing functional groups (e.g., carboxyl, hydroxyl), which promote chemisorption via hydrogen bonding or electrostatic interactions with negatively charged bacterial cell walls [50]. In contrast, maifanite primarily relies on cation bridging (e.g., Ca^2+^, Mg^2+^) for adsorption [13], while ceramsite’s inert, sintered surface confines it mostly to physical interception, resulting in the weakest performance [51].

A key observation was that for *Enterococcus*, which was present at lower concentrations in runoff, the adsorption capacities of all three media were similar and markedly lower. This dependency of removal efficiency on initial pathogen load and the dominant adsorption mechanism suggests that the EG system may have limited efficacy against low-concentration microbial pollution. These findings align with earlier studies on bioretention, in which biochar amendment and optimized media bed height were critical for significant pathogen removal. Consequently, effectively treating low-concentration pathogens likely requires specially designed systems, potentially through strategic media amendments and configuration optimization.

### 3.5. Integrated Pollutant Removal and Design Implications

**Figure 9:**
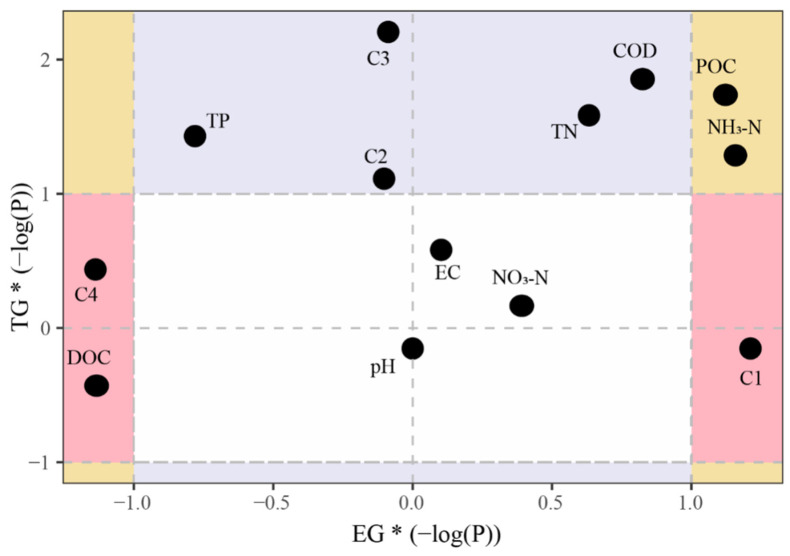
Quadrant analysis of the relationship between fecal indicator bacteria and environmental factors. Axes are scaled to the negative log-transformed *p*-values; greater absolute values correspond to higher statistical significance. Plot regions are defined as follows: light pink-significant correlation only with TG pathogen removal; purple-significant correlation only with EG pathogen removal; pale yellow—significant correlation shared by both systems; white—no significant correlation with either system. C1 represents protein-like components, C2 denotes fulvic-like components, C3 indicates humic-like components, and C4 corresponds to microbial by-product-like components.

Significance analysis (Figure 9) indicated that in the TG system, only the removal rates of DOC, C1, and C4 showed significant correlations with pathogen removal. This suggests that pathogen removal in TG relies more on the synchronous reduction of active DOM and associated microbial metabolic processes. Specifically, C4, representing microbial metabolites—often closely linked to microbial growth and antagonism—displayed a significant correlation. This implies that pathogen removal in TG may be achieved by enhancing microbial competition, predation, or metabolic inhibition, mechanisms that do not dominate in the EG system.

In contrast, in the EG system, only the removal rates of TP, C2 (fulvic-acid-like organics), C3 (humic-acid-like organics), TN, and COD correlated significantly with pathogen removal. This indicates that pathogen removal in EG is more governed by overall nutrient levels and structural changes in humified organic matter. Fulvic and humic acids possess strong complexation capacity and surface activity; their removal may alter the adsorption–desorption behavior and survival microenvironment of pathogens [52], thereby improving removal efficiency. Furthermore, the significant correlations with TN and COD suggest that pathogen removal in EG is more sensitive to system-load variations, likely exerting indirect effects by modulating redox conditions and microbial community composition.

In addition, removal rates of POC and NO_3_^−^-N were significantly correlated with pathogen removal in both the EG and TG systems, indicating that particulate organic matter and nitrate nitrogen act as common regulatory factors. POC removal may increase pathogen exposure and the probability of inactivation by weakening particle-mediated adhesion and shielding. Variations in NO_3_^−^-N likely reflect enhanced denitrification or other nitrogen-transformation processes, which are often accompanied by elevated microbial activity that can facilitate pathogen removal across systems [53]. Consequently, managing POC and NO_3_^−^-N dynamics could be an effective strategy to improve microbial removal performance in both system types. The TG system is driven more by active organic matter and microbial metabolism, while the EG system is regulated mainly by nutrients and humified organic matter. These findings establish a foundation for optimizing pathogen-control strategies according to process type. The EG system functions as an integrated treatment train, capitalizing on the distinct properties of each filler within a stratified configuration. Ceramsite acts as a roughing filter for solids and high-concentration DOM; maifanite serves as a secondary adsorber targeting specific DOM fractions and mid-size particles; and biochar operates as a polishing medium for dissolved nutrients, pathogens, and fine particulates. This cascaded configuration maximizes pollutant exposure to targeted removal pathways, thereby achieving synergistic performance beyond the reach of single-media systems such as conventional gravel gabions. Compared to other nature-based solutions (e.g., constructed wetlands or bioretention cells), the EG system offers a compact, modular design with clearly defined functional zones, which renders it a versatile alternative. To further enhance its efficacy against low-concentration pathogens and recalcitrant DOM fractions, modifications such as incorporating engineered biochar or extending hydraulic retention time could be employed to stimulate biological degradation.

It should be noted that this study was conducted using a single gabion unit subjected to ten consecutive operational cycles. Although each influent event was controlled independently, the internal conditions within the gabion matrix may have evolved progressively over time, potentially introducing dependencies between earlier and later cycles. As such, these cycles are more appropriately interpreted as a time-series representation of system performance rather than as independent experimental replicates [54]. While this design limits the applicability of certain parametric statistical tests that assume data independence, it offers valuable insight into the dynamic behavior and progressive maturation of the gabion system under repeated hydraulic and pollutant loading. This progressive evolution reveals potential shifts in the physical, chemical, and even biological micro-environments within the system, such as the initial accumulation of particulate matter and the subsequent gradual formation of biofilms [55,56]. These factors collectively influence the overall purification efficiency of the system. Therefore, considering this operational process as a time series facilitates a more realistic reflection of the continuous performance trajectory of the system in practical engineering applications.

Regarding the potential issue of clogging within the system, the interstitial voids between the stones may become progressively filled with intercepted materials, potentially leading to clogging. This phenomenon may initially lead to a progressive reduction in hydraulic conductivity, manifested as diminished water throughput and reduced treatment flow rates [57]. Over extended operation, clogging may also induce heterogeneous flow distribution within the system, leading to the formation of preferential flow paths or dead zones. Such conditions can result in inadequate contact between pollutants and the microbial communities or adsorption media, subsequently causing fluctuations or declines in overall removal efficiency [58]. Therefore, the long-term performance sustainability of the system warrants further investigation, with particular attention to the evolutionary patterns of internal porosity and permeability coefficients and their quantitative relationship with operational time. Future research should focus on developing and evaluating routine maintenance protocols—such as periodic backwashing, intermittent draining, or replacement of the surface stone layer—to mitigate clogging effects and ensure consistent treatment performance. Concurrently, it is advisable to employ predictive mathematical models to simulate the clogging process, enabling more precise determination of maintenance timing and intervention intensity.

## 4. Conclusions

In conclusion, this study demonstrates that an enhanced EG system with stratified functional fillers and integrated vegetation significantly improves the removal of organic pollutants and pathogens from stormwater compared to a traditional TG system.

The EG system achieved high removal efficiencies for COD (70.51%), NH_4_^+^–N (46.23%), TN (54.79%), POC (67.38–86.95%), and DOC (25.18–42.72%), along with notable reductions in FIB, including up to 45% for *E. coli*. Overall, the EG offers a compact, modular, and sustainable technology well-suited for riparian applications, providing a viable alternative to conventional bioretention systems for integrated stormwater quality improvement. To advance its application, subsequent efforts should focus on evaluating long-term performance, conducting field-scale validation, and optimizing filler media combinations to improve nutrient and pathogen removal across diverse hydraulic regimes.

## Figures and Tables

**Table 1:** Physicochemical properties of the tested samples.

Physicochemical Index	Soil
pH	8.12 ± 0.19
Electrical Conductivity (μS/cm)	166.60 ± 0.51
Ammonia Nitrogen (mg/g)	0.36 ± 0.01
Nitrate Nitrogen (g/g)	0.15 ± 0.01
Total Nitrogen (g/g)	2.00 ± 0.21
Total Phosphorus (g/g)	0.29 ± 0.01
Moisture Content (%)	11.46 ± 0.13
Organic Matter Content (%)	10.03 ± 0.16

**Table 2:** Primer and TaqMan probe sequences in this study.

Pathogenic Microorganisms	Function	Sequence (5′→3′)	Reference
Total coliforms	Forward primer	GTTGTAAAGCACTTTGAGTGGTGAGGAAGG	[16]
	Reverse primer	GCCTCAAGGGCACAACCTCCAAG	
Fecal coliforms	Forward primer	AGAGTTTGATCCTGGCTCAG	[19]
	Reverse primer	CGGGTAACGTCAATGAGCAAA	
*Escherichia coli*	Forward primer	GGGGCGGTGACGCAG	[20]
	Reverse primer	CCTGGTGAGTCGGAATGGTG	
	Probe †	CGATGATGCGCGGCG	
*Enterococcus* spp.	Forward primer	TCTCATCGGCTCCTACCTATC	[16]
	Reverse primer	AAGCTGTGGACTACACCATTAG	

Note: † The TaqMan probe was labeled with 5′-FAM (6-carboxyfluorescein) and 3′-MGB-NFQ (minor groove binder–non-fluorescent quencher).

## Data Availability

The data presented in this study are available upon request from the corresponding author.
